# Biomass-Based Hydrothermal Carbons for the Contaminants Removal of Wastewater: A Mini-Review

**DOI:** 10.3390/ijms24021769

**Published:** 2023-01-16

**Authors:** Yuanyuan Wang, Yuan Xu, Xintian Lu, Kefeng Liu, Fengfeng Li, Bing Wang, Qiang Wang, Xv Zhang, Guihua Yang, Jiachuan Chen

**Affiliations:** 1 State Key Laboratory of Biobased Material and Green Papermaking, Qilu University of Technology (Shandong Academy of Sciences), Jinan 250353, China; 2 Liaoning Key Laboratory of Lignocellulose Chemistry and Biomaterials, Dalian Polytechnic University, Dalian 116034, China

**Keywords:** biomass, hydrothermal carbon, adsorbents, heavy metals, organic dyes, adsorption removal

## Abstract

The preparation of adsorbents with eco-friendly and high-efficiency characteristics is an important approach for pollutant removal, and can relieve the pressure of water shortage and environmental pollution. In recent studies, much attention has been paid to the potential of hydrothermal carbonization (HTC) from biomass, such as cellulose, hemicellulose, lignin, and agricultural waste for the preparation of adsorbents. Hereby, this paper summarizes the state of research on carbon adsorbents developed from various sources with HTC. The reaction mechanism of HTC, the different products, the modification of hydrochar to obtain activated carbon, and the treatment of heavy metal pollution and organic dyes from wastewater are reviewed. The maximum adsorption capacity of carbon from different biomass sources was also evaluated.

## 1. Introduction

The threat posed by hazardous contaminants is increasing, and in recent years, heavy metal ions and organic dyes have contributed to widespread water contamination. Numerous treatment techniques, including adsorption, reduction reactions, membrane processes, chemical precipitation, ion exchange, and bioremediation, etc., have been developed by researchers [1,2,3,4,5,6]. Among these methods, adsorption has received more and more attention owing to its extensive application potential, simple operation, and high repeatability [7,8]. Therefore, the development of effective preparation techniques and low-cost, high-performance adsorbents is crucial for the treatment of wastewater.

During the adsorption process, physical adsorption and chemical adsorption are the two main methods to remove organic dyes and heavy metal ions from wastewater. The chemical adsorption is that adsorbent can bond with contaminants via chemical bond, and chemical adsorption is strongly tied to active groups including hydroxyl, carboxyl, etc., whereas physical adsorption is mainly related to the forces between molecules (mainly electrostatic attraction) [7,9,10]. It is worth noting that carbon materials have emerged as a viable adsorbent contender owing to their huge pore volume, abundance of active sites, robust mechanical capabilities, and excellent thermostability [11,12]. Currently, various adsorbents have been prepared from abundant, economical, and green resources, mainly including industrial and agricultural waste [13,14].

The hydrothermal carbonization (HTC) method is a thermo-chemical production strategy that takes place in a watery environment, at 180–250 °C, and under autogenous saturated vapor conditions [15,16]. The primary product of the HTC is hydrochar, which can be used in adsorption, energy, agriculture, and the environment as a precursor to produce activated carbons [16]. Because of the abundance of oxygen-containing groups generated under HTC and given conditions, biomass-based hydrothermal carbons recently showed great potential in the field of pollutant adsorption [17,18]. Therefore, biochar is deemed as a promising material for adsorbing pollutant in wastewater due to the low-cost of raw materials, availability from a variety of sources, and effective adsorption capabilities.

Lignocellulosic biomass, as one of the most abundant natural resources, and is mainly composed of cellulose, hemicellulose, and lignin [16]. Typically, cellulose and hemicelluloses are partially or completely degraded during the HTC process, yielding hydrochar with a high lignin content. The conversion of forestry and agricultural wastes into cost-effective pollutant adsorbents is a good strategy for both waste utilization and control of wastewater [19]. The high-value utilization of biomass reduces the cost of hydrothermal carbon preparation. However, the functionality and surface properties of the carbon generated directly from the HTC are unsatisfactory for the removal of pollutants from wastewater, necessitating the development of simple and efficient procedures to improve their functionality. This review summarizes the latest studies and developments on HTC mechanisms and carbon material properties obtained from cellulose, hemicellulose, lignin, and crude biomass reported, and focuses especially on the structure-function relationship of activated carbon. Therefore, this review will pave the way for innovative design directions and apply novel biomass-based material to excellent adsorption performance.

## 2. The HTC of Biomass

**Figure 1:**
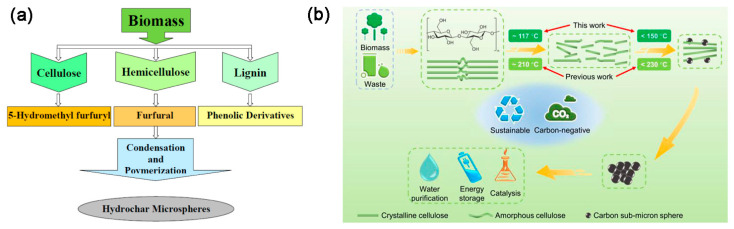
(**a**) Flowchart depicting hydrochar formation. Reprinted with permission from Ref. [20]. Copyright 2020 Springer Nature. (**b**) Schematic illustration of carbon sub-micron spheres from the low-temperature DTPH system of cellulose-based feedstocks. Reprinted with permission from Ref. [21]. Copyright 2022 Springer Nature.

**Figure 2:**
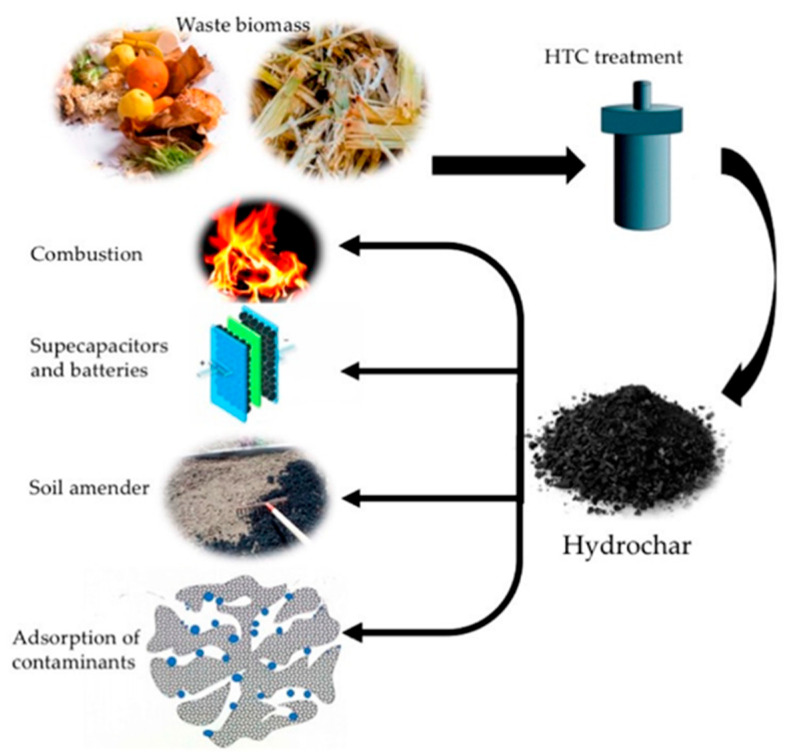
The potential applications of waste biomass. Reprinted with permission from Ref. [22]. Copyright 2020 MDPI.

Cellulose, with its molecular formula of (C_6_H_10_O_5_)_n_, is a water-insoluble polymer linked by glycosidic bonds. The hemicellulose is a complex glycan composed of various sugar groups and uronic acid groups, and abundant free hydroxyl groups are distributed along the chains of hemicellulose molecules. Lignin is a three-dimensional, amorphous aromatic polymer consisting of various structural units and links, mainly those including aromatic rings, aryl ether bonds, and different hydroxyl radicals, etc. [6]. Under the same HTC conditions, the lignocellulosic components typically degraded in the following order: hemicellulose > cellulose > lignin [16]. Although the types of biomass have significant influence on the quantity and yield of hydrochar, the basic process of hydrochar formation during conventional HTC in a typical batch reactor, is shown in Figure 1a [20]. In recent study, a decoupled temperature and pressure hydrothermal (DTPH) system has been developed, and cellulose was heated and degraded at a constant pressure and low temperature during the DTPH process, resulting in the rapid preparation of carbon sub-micron spheres (Figure 1b) [21]. In addition, as shown in Figure 2, hydrochar from biomass has a variety of potential applications that can be used in combustion, supercapacitors, batteries, soil amender, adsorption of contaminants, and so on [22].

**Figure 3:**
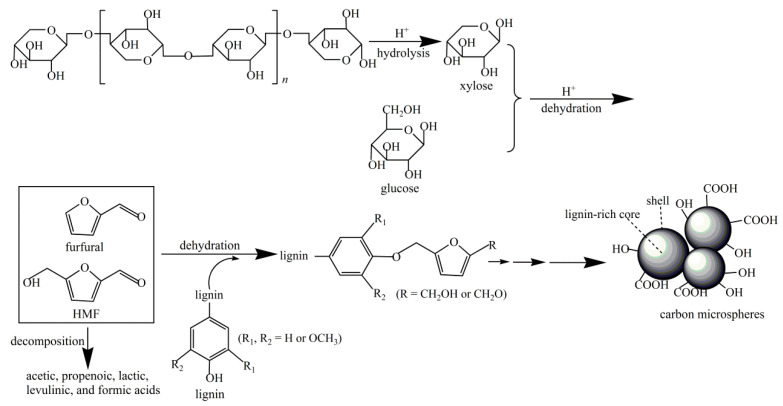
The preparation mechanism of CM-PHL. Reprinted with permission from Ref. [23]. Copyright 2019 Elsevier.

**Figure 4:**
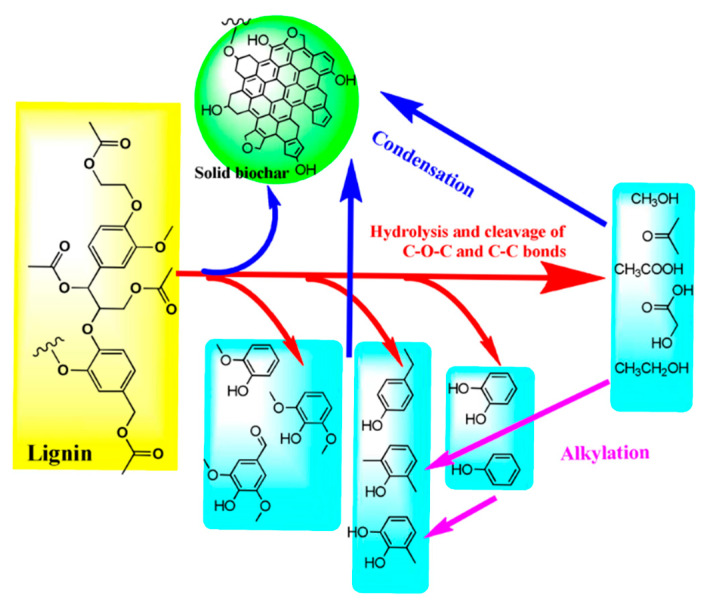
The mechanism of lignin HTC. Reprinted with permission from Ref. [29]. Copyright 2015 Royal Society of Chemistry.

Typically, the formation process of hydrochar depends on the type of biomass being used. The reaction pathways of the main constituents of biomass are simplified as follows: (1) Cellulose successively hydrolyzes into oligosaccharides and glucose monosaccharide and then generates fructose. After that, they can further dehydrate and generate hydroxymethyl furfural (HMF). It would polymerize into biochar or possibly rehydrate into formic acid and levulinic acid with further processing. However, in Yu’s research, it demonstrated that hydrochar was produced from the original cellulose directly in the DTPH process of cellulose [21]. The intermediates were produced from cellulose through intramolecular and intermolecular rearrangement. After that, the obtained intermediates formed an aromatic structure, which was used to produce char. (2) The hydrolysis of hemicelluloses would firstly generate oligosaccharides and then monomeric xylose. Subsequently, the obtained monomeric xylose can be used to produce furfural by further dehydration. Wang et al. prepared hydrochar using hemicellulose-rich pre-hydrolysis liquor (PHL) via HTC, and further modified with acrylic acid and sulfuric acid to prepare carbon microspheres (CM-PHL) [23]. The result showed that the adsorption performance of CM-PHL reached as high as 701.3 mg/g for methylene blue. As shown in Figure 3, the high H^+^ concentration during the HTC process would be beneficial to the subsequent hydrolysis and dehydration, which could successively produce monosaccharides and furan compounds. Generally, CM-PHL can be produced from above furan compounds via condensation or polymerization. Meanwhile, it will be inclined to form CM-PHL particles with large size from furan compounds by reacting with lignin and gathering carbon nuclei. (3) Hydrolysis of lignin polymers can produce several phenolic compounds and corresponding polycondensates [24]. In the HTC of lignin, the cleavage of linkages, especially of β-O-4 ether bonds and C-C bonds, is one of the most important reactions [25]. Meanwhile, some condensation, demethoxylation, and alkylation reactions will occur [26,27]. However, the bonds in the aromatic rings usually retain stability during HTC [28]. The mechanism of lignin HTC was presented in Figure 4 [29]. It was found that the required temperature of cellulose is generally higher than that of xylan, and the specific surface area of hydrochar from cellulose is arrestingly higher than that from xylan [30].

Although many efforts have been made to reveal the effects of HTC on the individual lignocellulosic components, it is different from the HTC of the crude biomass because of the multiple connections between the components [16]. Yu et al. proposed the evolution pathways from real biomass feedstocks to hydrochar [31]. It was reported that small pieces with the surface modification of C-O cleavage were first obtained from room temperature to 100 °C. From 100 to 200 °C, the obtained small pieces can be used to produce carbon spheres with aromatization due to the process of dehydration. Subsequently, nanospheres were formed from the decomposition of microspheres via aromatization (200–300 °C).

## 3. Hydrochar as Adsorbents for Pollution Control from Wastewater

Hydrochars received a substantial infusion of oxygen-containing functional groups during the HTC process, which is favorable for the adsorption of pollutants. The adsorbents produced by HTC of low-cost biomass sources may provide a promising way to remediate wastewater economically and effectively of both organic and inorganic pollutants.

### 3.1. Hydrochar for Heavy Metal Ion Adsorption

The pentosan has been used to produce carboxyl-rich carbon microspheres (CSp) via HTC with no other additives [17]. The obtained CSp has good dispersion and thermal stability, and contains a large number of oxygen-containing groups. This makes the CSp have a good adsorption capacity for Pb (II) (380.1 mg/g) and Cd (II) (100.8 mg/g). In order to improve the adsorption performance of hydrothermal carbon, some additives were added in the hydrothermal process to introduce functional groups such as carboxyl, hydroxyl, and nitrogen-contained on the surface of hydrothermal carbon to increase the chemical adsorption capacity [7,32]. Hemicelluloses may be hydrolyzed, dehydrated, and aromatized into carbon spheres at a very low temperature by adding sulfuric acid during the HTC process [33]. Additionally, some studies have demonstrated that the addition of phloroglucinol significantly increased the yield of the carbon spheres produced from mono-saccharides [34]. In Demir-Cakan’s research, by carbonizing glucose hydrothermally in one step with acrylic acid present, numerous carboxylate groups were given to the hydrothermal carbon [35]. The hydrothermal carbon exhibited micrometer-sized “raspberry”-like structures, and the adsorption performance of hydrothermal carbon for Pb (II) was reached up to 351.4 mg/g under the optimal conditions. In the presence of thioglycolic acid, ethylenediamine, and acrylic acid, Liu et al. successfully prepared glucose based carbons via the modification of thiol, amino, and carboxyl groups [36]. The adsorption capacities reached as high as 171.23, 94.25, and 15.41 mg/g for Cr (VI), which were higher than those of hydrothermal carbon without functionalization (13.00 mg/g). In Wei’s research, factory waste hemicelluloses were selected as raw material to fabricate nitrogen doping hydrothermal carbon with abundant Schiff base structures (NHTC-SBS) via HTC in ammonia solution [7]. The maximum adsorption capacity of NHTC-SBS towards Cr (VI) reached 349.6 mg/g, which mainly relied on electrostatic attraction, reduction of Cr (VI) to Cr (III), and chelation. Tian et al. successfully produced N-doped biochar from purified lignin (PL) and black liquor (BL) via a simple one-step HTC, and was applied in the adsorption of Cr (VI) [32]. The result showed that the maximum adsorption performance of N-BL-180 was 859.43 mg/g, whereas that of NPL-150 was 583.77 mg/g. It is obvious that the introduction of functional groups, especially N-containing group, can significantly enhance the heavy metal ion adsorption capacity of biochar.

Compared with conventional heating techniques, microwave heating has various benefits [37,38]. Previous research has demonstrated that microwave assisted hydrothermal process was a viable alternative for the quick generation of hydrochar. In Li’s research, hydrochars were fabricated from rice straw via microwave hydrothermal synergy process, and the equilibrium of HTC reactions was reached rapidly [37]. The hydrochar with abundant oxygen functional groups was obtained, and was used for adsorption of heavy metal. It was found that the maximum adsorption amounts of Cu (II) and Zn (II) reached up to 144.9 and 112.8 mg/g, respectively.

Besides, other biomass-based hydrothermal carbon with excellent adsorption abilities was shown in Table 1. Each biomass-based hydrothermal carbon presents its own features. The adsorption capacity of hydrothermal carbon can be significantly improved by the functionalization with carboxyl, hydroxyl, and nitrogen.

### 3.2. Hydrochars for Organic Dyes Adsorption

Because of its excellent properties, hydrothermal carbon can be used to adsorb dyes from water. In general, methylene blue (MB) was adsorbed on carbon through hydrogen-bond formation, electrostatic interaction, π-π electron dispersion, and electron donor-acceptor relationships [43]. Besides, the adsorbing ability of carbon for MB was influenced by the carbon pore structure. In our previous research, the carbon microspheres were prepared from hemicellulose-rich PHL through the HTC process [23]. The lignin and sulfuric acid notably increased the carbon microspheres yield and their sizes. Additionally, the adsorption capacities of carbon microspheres were improved after functionalized with acrylic acid, and the highest adsorption capacities for MB was 701.3 mg/g. Through adding acrylic acid and ammonium persulphate in the HTC process and the following sodium hydroxide solution activation, Lv et al. successfully prepared hydrochar (AAHC) with carboxylate groups [44]. The obtained AAHC displayed larger pore volumes, higher BET surfaces, and more carboxylate groups than that prepared without using ammonium persulfate, demonstrating that even a small amount of ammonium persulfate could play a vital role as a HTC-based radical initiator. Benefiting from the above advantages, the maximum adsorption capacity of AAHC toward MB reached 717.78 mg/g, indicating that AAHC might be a potential option for MB adsorption from aqueous solutions. Thus, it is plausible that the addition of acrylic acid in the HTC process can improve the MB adsorption capacity of hydrochar.

## 4. Hydrochar Modification to Obtain Activated Carbon

A simple HTC treatment is difficult to produce a sufficiently porous carbon. To increase the adsorption capacity of hydrochar, many methods, such as alkalinity modification, acid modification as well as metal salts treatment, were adopted to modulate physicochemical properties (e.g., functional groups, porosity, and surface charge) [45]. Danish et al. reported that the pore structure, surface area, and functionality were significantly influenced by the types of gas and chemicals used during the activation process, and the adsorption capacity of activated carbon ultimately was affected [46].

Acid modification, such as using hydrochloric acid, nitric acid, or phosphoric acid, is mostly used to introduce acid functional group, alter the surface area, and thus increase the ability of activated carbon to remove pollutants [45]. For example, the hydrophobic adsorption sites of reed based biochar were significantly increased thanks to the treatment of hydrochloric acid (1 mol/L), while the surface area was enhanced from 58.75 to 88.35 m^2^/g and ash content was decreased from 29.5 to 11.8% [47]. Fernandez et al. reported that the mesopore volume and specific surface areas of orange peel-derived carbon were obviously enhanced (from 0.025 cm^3^/g and 117 m^2^/g to 0.102 cm^3^/g and 618 m^2^/g, respectively) after chemical activation with H_3_PO_4_, and the adsorption performance was significantly improved [48].

The main purpose of metal salts treatment during the HTC process is to speed up the reaction rate, regulate pore structure, increase the surface area, and provide more active sites for the adsorption of pollutants [49]. Nguyen’s research found that the microporosity fraction, active surface area and active sites of hydrothermal carbon obtained from algal were significantly increased after activating with ZnCl_2_, and thus the high adsorption capacity (400 mg/g) for ciprofloxacin was achieved [50].

**Figure 5:**
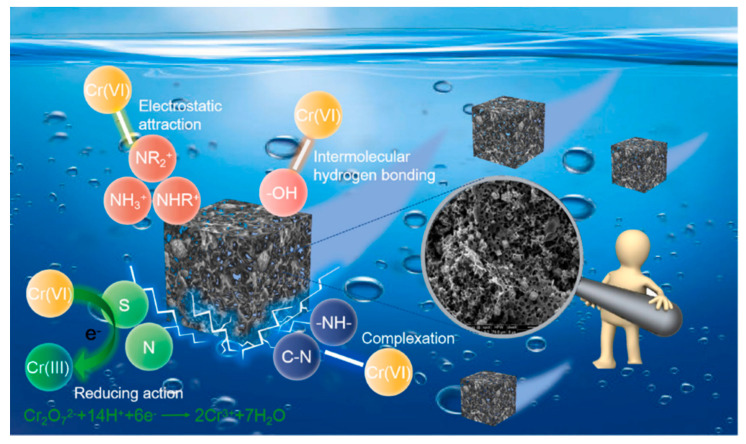
Adsorption mechanism of Cr (VI) by L-HPC. Reprinted with permission from Ref. [51]. Copyright 2022 Elsevier.

The purpose of alkaline treatment (e.g., potassium hydroxide and sodium hydroxide) is to increase the specific surface areas and oxygen-containing functional groups. As an example, the industrial alkali lignin was used to fabricate hierarchical porous carbon-based adsorbent (L-HPC) to adsorption of Cr (VI) through a combined process of hydrothermal and alkali activation [51]. Some three-dimensional connected channels were formed and abundant active groups, e.g., N and O containing groups were generated during the KOH treatment. Meanwhile, the specific surface area and pore volume of L-HPC improved from 3.74 m^2^/g, 0.001 cm^3^/g to 749.16 m^2^/g, 0.227 cm^3^/g, and the average pore size decreased from 9.63 to 3.56 nm, which will significantly contribute to the removal of Cr (VI). The adsorption capacity reaches 887.8 mg/g and L-HPC has good regenerative ability, indicating that L-HPC presents great potential in heavy metal adsorption of wastewater. In addition, the adsorption mechanism of L-HPC was investigated and divided into four types: electrostatic attraction, reduction, complexation, and hydrogen force (Figure 5).

Liang et al., prepared nitrogen-doped carbon spheres (CSs-N) from urea and glucose via a hydrothermal process [52]. To further improve the properties, CSs-N was then activated by KOH to obtain porous carbon material (PCM-N). It was found that PCM-N had a high specific surface area of 1600.67 m^2^/g and maximum adsorption capacity of 402.9 mg/g for Cr (VI). Mesoporous activated carbon (COSHTC) was prepared using a hydrochar derived from coconut shell waste through HTC and NaOH chemical activation process, which exhibited an optimum performance because of its mesoporosity, pore volume 0.441 of cm^3^/g, and surface area of 876.14 m^2^/g [53]. The adsorption capacity of MB was 200 mg/g at 30 °C. In Tan’s research, lignin-derived porous carbons (LPC) were fabricated through hydrothermal process and KOH activation and then was used to evaluate MB adsorption [54]. It was found that LPC possessed a large number of active sites and a high specific surface area (3382.32 m^2^/g). The maximum adsorption capacity of MB was 1119.18 mg/g. Comparing the properties of the two activated carbons, it can be found that the activated carbon with high specific surface area has strong adsorption capacity.

Additionally, the biomass-based activated carbon with excellent adsorption capacities reported in recent years was shown in Table 2, and summarized the properties of activated carbon. It can be seen from Table 2 that the adsorption capacity of activated carbon is affected by raw material, specific surface area, pore size, and so on.

Compared with hydrochar, the activated carbon materials obtained from biomass have both superiorities and inferiorities. In common, a more complicated process is required for activated carbon preparation than that of hydrochar. Meanwhile, the characteristic of carbon materials is influenced by many parameters, including raw material features, activator, dosages, pyrolysis temperatures and processes. Generally, activated carbon materials exhibit more abundant pores and higher surface area compared with hydrochar [59], which makes it more suitable for adsorption.

Although the activated carbon has a large specific surface area and strong adsorption capacity, it needs to be carbonized at high temperature in the activation and consumes higher energy. The adsorption capacity of hydrothermal carbon to pollutants can be significantly increased by impregnating hydrothermal carbon with aqueous solutions of NaOH, which can reduce the energy consumption compared with the high temperature carbonization. In Song’s research, hydrothermal carbon nanospheres (CNS) were prepared from glucose, and activated with aqueous solutions of NaOH [60]. The results indicated that the surface of CNS activated by impregnating with NaOH solution was enriched with -OH and -COO^−^, and the morphology, surface area and pore volume were not noticeable changes. CNS activated by NaOH exhibited excellent adsorption capacity of 152 mg/g for silver ions. According to the results of FTIR characterized of carbon materials, it indicated that NaOH reacted with the lactone and carboxyl groups present on the surface of CNS to increase the amount of -OH and -COO^−^ [60]. Our previous study found the same result that the adsorption capacity of hydrothermal carbon for Pb (II) and MB increased obviously via the activation of aqueous solutions of NaOH [23].

## 5. Conclusions and Future Perspectives

In recent years, different adsorption materials have been developed and synthesized to remove contaminants from wastewater. Among them, the hydrothermal carbon-based adsorbent an efficient adsorbent for the removal of organic and heavy metal contaminants from wastewater because of its incomparable advantages, such as the low price of raw materials, wide range of sources, simple and environmentally friendly preparation process, and good adsorption performance. At present, the preparation of hydrothermal carbon adsorbents have the following problems: (1) Most hydrothermal carbon obtained by HTC has a small surface area, low porosity, and insufficient active groups on the surface. In order to improve the physical adsorption capacity of carbon materials, it is required to increase the specific surface area and porosity. Additionally, the implantation of other active groups on the surface of hydrothermal carbon, such as carboxyl, hydroxyl, and the nitrogen-containing group, is one of the most effective methods to improve the chemical adsorption ability. Thus, the regulation of the HTC process and the selection of appropriate functional reagents are important factors to improve the adsorption performance. (2) There are more liquids obtained from HTC, and the utilization of the whole components of the liquid is an important problem. In general, 3–10 times the mass of biomass water is required in the HTC process [61]. Then, a large amount of waste liquid is obtained after HTC, which contains glucose, fructose, xylose, furfural, 5-hydroxyl methyl furfural, acetic acid, formic acid, lactic acid, phenol, and so on, resulting from the degradation of some substances [62]. However, there is little research or use of aqueous fraction resulting from HTC. In order to make the process more environmentally friendly and economically feasible, it is necessary to find effective methods to treat or use aqueous fraction resulting from HTC. (3) The mechanism of HTC of biomass is not clear, which seriously limits the directional preparation and high-value utilization of HTC products. (4) The study on adsorption mechanism of hydrothermal carbon with various functional groups and structures is deficient, and the effect mechanisms of exterior factors on adsorption capacity, such as initial concentrations of adsorbate, pH, ionic strength, temperature, and adsorption time, etc., is insufficient. Therefore, it is necessary to further develop the bio-based functional hydrothermal carbon as adsorbent materials, so that they will play an important role in water pollution control.

## Figures and Tables

**Table 1:** Adsorption capacity of biomass-based hydrothermal carbon.

Raw Materials	Additives	Functional Groups	Contaminants	Adsorption Capacity (mg/g)	References
Hemicelluloses	Ammonia solution	-OH, -COOH, -C=O, C-N, N-H	Cr (VI)	74.60	[39]
Hemicellluloses-rich pre-hydrolysis liquor	Acrylic acid, sulfuric acid	-OH, -COOH	Pb (II)	273.4	[23]
Eupatorium adenophorum	-	-OH, -COOH, -O-, C=C	Pb (II)	164.68	[40]
Glucose	Acrylic acid	-OH, -COOH, C=C	Cd (II)	88.8	[35]
Bamboo	Polyvinyl chloride	-OH, O-C=O, C-O	MB	258.97	[18]
Bamboo	Maleylated	COO-	MB, Cd (II)	1155.57, 90.99	[41]
Cassava slag	-	-OH, -COOH, C-O-C	Rhodamine B	105.3	[13]
Glucose	Anionic polyacrylamide	C=C, -COO^-^	MB	348.1	[42]

**Table 2:** Source of biomass, chemical activator and structural properties of activated carbon and adsorption capacity.

Biomass	Activator	Contaminants	Properties of Activated Carbon			References
			* S_BET_(m^2^/g)	Pore Diameter (nm)	Adsorption Capacity (mg/g)	
Tea	NaOH	MB	368.92	23.02	487.4	[55]
Rattan furniture wastes	NaOH	MB	1135	3.55	359	[56]
Sucrose	KOH	MB	1534	2.0	704.2	[57]
Sewage sludge and coconut shell	KOH	MB	873.54	2.51	623.37	[58]

* The S_BET_ refers to BET specific surface areas.

## Data Availability

Not applicable.

## References

[1] Pan F., Cao Z., Zhao Q., Liang H., Zhang J. (2014). Nitrogen-doped porous carbon nanosheets made from biomass as highly active electrocatalyst for oxygen reduction reaction. J. Power Sources.

[2] Subedi N., Lahde A., Abu-Danso E., Iqbal J., Bhatnagar A. (2019). A comparative study of magnetic chitosan (Chi@Fe_3_O_4_) and graphene oxide modified magnetic chitosan (Chi@Fe_3_O_4_GO) nanocomposites for efficient removal of Cr(VI) from water. Int. J. Biol. Macromol..

[3] Li S., Liu L., Yu Y., Wang G., Zhang H., Chen A. (2017). Fe_3_O_4_ modified mesoporous carbon nanospheres: Magnetically separable adsorbent for hexavalent chromium. J. Alloys Compd..

[4] Duran U., Coronado-Apodaca K.G., Meza-Escalante E.R., Ulloa-Mercado G., Serrano D. (2018). Two combined mechanisms responsible to hexavalent chromium removal on active anaerobic granular consortium. Chemosphere.

[5] Palansooriya K.N., Yang Y., Tsang Y.F., Sarkar B., Hou D., Cao X., Meers E., Rinklebe J., Kim K.H., Ok Y.S. (2019). Occurrence of contaminants in drinking water sources and the potential of biochar for water quality improvement: A review. Crit. Rev. Environ. Sci. Technol..

[6] Zhang W., Duo H., Li S., An Y., Chen Z., Liu Z., Ren Y., Wang S., Zhang X., Wang X. (2020). An overview of the recent advances in functionalization biomass adsorbents for toxic metals removal. Colloid Interface Sci..

[7] Wei Y., Wang H., Zhang X., Liu C. (2021). Ammonia-assisted hydrothermal carbon material with schiff base structures synthesized from factory waste hemicelluloses for Cr (VI) adsorption. J. Environ. Chem. Eng..

[8] Sun Y., Wang T., Sun X., Bai L., Han C., Zhang P. (2021). The potential of biochar and lignin-based adsorbents for wastewater treatment: Comparison, mechanism, and application—A review. Ind. Crops Prod..

[9] Wan J., Liu F., Wang G., Liang W., Peng C., Zhang W., Lin K., Yang J. (2021). Exploring different mechanisms of biochars in removing hexavalent chromium: Sorption, reduction and electron shuttle. Bioresour. Technol..

[10] Sun Y., Liu C., Zan Y., Miao G., Wang H., Kong L. (2018). Hydrothermal carbonization of microalgae (*Chlorococcum* sp.) for porous carbons with high Cr(VI) adsorption performance. Appl. Biochem. Biotechnol..

[11] Chen M., Li J., Zhang J., Ma Y., Dong H., Li W., Bekyarova E., Al-Hadeethi Y.F., Chen L., Hedhili M.N. (2021). Evolution of cellulose acetate to monolayer graphene. Carbon.

[12] Gupta V.K., Saleh T.A. (2013). Sorption of pollutants by porous carbon, carbon nanotubes and fullerene—An overview. Environ. Sci. Pollut. Res..

[13] Wu J., Yang J., Huang G., Xu C., Lin B. (2020). Hydrothermal carbonization synthesis of cassava slag biochar with excellent adsorption performance for Rhodamine B. J. Clean. Prod..

[14] Hou Y., Huang G., Li J., Yang Q., Huang S., Cai J. (2019). Hydrothermal conversion of bamboo shoot shell to biochar: Preliminary studies of adsorption equilibrium and kinetics for rhodamine B removal. J. Anal. Appl. Pyrol..

[15] Xiao K., Liu H., Li Y., Yi L., Zhang X., Hu H., Yao H. (2018). Correlations between hydrochar properties and chemical constitution of orange peel waste during hydrothermal carbonization. Bioresour. Technol..

[16] Antero R.V.P., Alves A.C.F., de Oliveira S.B., Ojala S.A., Brum S.S. (2020). Challenges and alternatives for the adequacy of hydrothermal carbonization of lignocellulosic biomass in cleaner production systems: A review. J. Clean. Prod..

[17] Wu Q., Li W., Liu S. (2014). Carboxyl-rich carbon microspheres prepared from pentosan with high adsorption capacity for heavy metal ions. Mater. Res. Bull..

[18] Li H.Z., Zhang Y.N., Guo J.Z., Lv J., Huan W., Li B. (2021). Preparation of hydrochar with high adsorption performance for methylene blue by co-hydrothermal carbonization of polyvinyl chloride and bamboo. Bioresour. Technol..

[19] Koushkbaghi S., Jafari P., Rabiei J., Irani M., Aliabadi M. (2016). Fabrication of PET/PAN/GO/Fe_3_O_4_ nanofibrous membrane for the removal of Pb (II) and Cr (VI) ions. Chem. Eng. J..

[20] Sharma R., Jasrotia K., Singh N., Ghosh P.Y., Srivastava S., Sharma N.R., Singh J., Kanwar R., Kumar A. (2020). A comprehensive review on hydrothermal carbonization of biomass and its applications. Chem. Afr..

[21] Yu S., Dong X., Zhao P., Luo Z., Sun Z., Yang X., Li Q., Wang L., Zhang Y., Zhou H. (2022). Decoupled temperature and pressure hydrothermal synthesis of carbon sub-micron spheres from cellulose. Nat. Commun..

[22] Maniscalco M.P., Volpe M., Messineo A. (2020). Hydrothermal carbonization as a valuable tool for energy and environmental applications: A review. Energies.

[23] Wang Y., Cao X., Sun S., Zhang R., Shi Q., Zheng L., Sun R. (2019). Carbon microspheres prepared from the hemicelluloses-rich pre-hydrolysis liquor for contaminant removal. Carbohyd. Polym..

[24] Stemann J., Putschew A., Ziegler F. (2013). Hydrothermal carbonization: Process water characterization and effects of water recirculation. Bioresour. Technol..

[25] Ehara K., Saka S., Kawamoto H. (2002). Characterization of the lignin-derived products from wood as treated in supercritical water. J. Wood Sci..

[26] Barbier J., Charon N., Dupassieux N., Loppint-Serani A., Mahé L., Ponthus J., Courtiade M., Ducrozet A., Quoineaud A., Cansell F. (2012). Hydrothermal conversion of lignin compounds. A detailed study of fragmentation and condensation reaction pathways. Biomass Bioenerg..

[27] Kang S., Li X., Fan J., Chang J. (2011). Classified separation of lignin hydrothermal liquefied products. Ind. Eng. Chem. Res..

[28] Wang H.J., Zhao Y., Wang C., Fu Y., Guo Q. (2009). Theoretical study on the pyrolysis process of lignin dimer model compounds. Acta Chim. Sin..

[29] Liu W.J., Jiang H., Yu H.Q. (2015). Thermochemical conversion of lignin to functional materials: A review and future directions. Green Chem..

[30] Sheng K., Zhang S., Liu J., E S., Jin C., Xu Z., Zhang X. (2019). Hydrothermal carbonization of cellulose and xylan into hydrochars and application on glucose isomerization. J. Clean. Prod..

[31] Yu S., Yang X., Zhao P., Li Q., Zhou H., Zhang Y. (2022). From biomass to hydrochar: Evolution on elemental composition, morphology, and chemical structure. J. Energy Inst..

[32] Tian Y., Yin Y., Liu H., Zhou H. (2022). One-step hydrothermal carbonization of amine modified black liquor and lignin for efficient Cr (VI) adsorption. J. Water Process Eng..

[33] Wang Y., Yang R., Li M., Zhao Z. (2015). Hydrothermal preparation of highly porous carbon spheres from hemp (*Cannabis sativa* L.) stem hemicellulose for use in energy-related applications. Ind. Crops Prod..

[34] Ryu J., Suh Y.W., Suh D.J., Ahn D. (2010). Hydrothermal preparation of carbon microspheres from mono-saccharides and phenolic compounds. Carbon.

[35] Demir-Cakan R., Baccile N., Antonietti M., Titirici M. (2009). Carboxylate-rich carbonaceous materials via one-step hydrothermal carbonization of glucose in the presence of acrylic acid. Chem. Mater..

[36] Liu L., Cai W., Dang C., Han B., Chen Y., Yi R., Fan J., Zhou J., Wei J. (2020). One-step vapor-phase assisted hydrothermal synthesis of functionalized carbons: Effects of surface groups on their physicochemical properties and adsorption performance for Cr (VI). Appl. Surf. Sci..

[37] Li Y., Tsend N., Li T.K., Liu H., Yang R., Gai X., Wang H., Shan S. (2019). Microwave assisted hydrothermal preparation of rice straw hydrochars for adsorption of organics and heavy metals. Bioresour. Technol..

[38] Zhang J., An Y., Borrion A., He W., Wang N., Chen Y., Li G. (2018). Process characteristics for microwave assisted hydrothermal carbonization of cellulose. Bioresour. Technol..

[39] Wei Y., Chen W., Liu C., Wang H. (2021). Facial synthesis of adsorbent from hemicelluloses for Cr (VI) adsorption. Molecules.

[40] Liu D., Tang Y., Li J., Hao Z., Zhu J., Wei J., Liu C., Dong L., Jia B., Chen G. (2021). Eupatorium adenophorum derived adsorbent by hydrothermal-assisted HNO_3_ modification and application to Pb^2+^ adsorption. J. Environ. Chem. Eng..

[41] Li B., Guo J., Lv K., Fan J. (2019). Adsorption of methylene blue and Cd (II) onto maleylated modified hydrochar from water. Environ. Pollut..

[42] Zhang Z., Wu G., Xu Z., Wu S., Gu L. (2018). Adsorption of Methyl Blue onto uniform carbonaceous spheres prepared via an anionic polyacrylamide-assisted hydrothermal route. Mater. Chem. Phys..

[43] Santoso E., Ediati R., Kusumawati Y., Bahruji H., Sulistiono D.O., Prasetyoko D. (2020). Review on recent advances of carbon based adsorbent for methylene blue removal from waste water. Mater. Today Chem..

[44] Lv B.W., Xu H., Guo J.Z., Bai L.Q., Li B. (2022). Efficient adsorption of methylene blue on carboxylate-rich hydrochar prepared by one-step hydrothermal carbonization of bamboo and acrylic acid with ammonium persulphate. J. Hazard. Mater..

[45] Wang J., Wang S. (2019). Preparation, modification and environmental application of biochar: A review. J. Clean. Prod..

[46] Danish M., Ahmad T., Hashim R., Said N., Akhtar M.N., Mohamad-saleh J., Sulaiman O. (2018). Comparison of surface properties of wood biomass activated carbons and their application against rhodamine B and methylene blue dye. Surf. Interfaces.

[47] Peng P., Lang Y.H., Wang X.M. (2016). Adsorption behavior and mechanism of pentachlorophenol on reed biochars: pH effect, pyrolysis temperature, hydrochloric acid treatment and isotherms. Ecol. Eng..

[48] Fernandez M.E., Ledesma B., Román S., Bonelli P.R., Cukierman A.L. (2015). Development and characterization of activated hydrochars from orange peels as potential adsorbents for emerging organic contaminants. Bioresour. Technol..

[49] Nirmaladevi S., Palanisamy P.N. (2021). Adsorptive behavior of biochar and zinc chloride activated hydrochar prepared from Acacia leucophloea wood sawdust: Kinetic equilibrium and thermodynamic studies. Desalin. Water Treat..

[50] Nguyen T.B., Truong Q.M., Chen C.W., Doong R.A., Chen W.H., Dong C.D. (2022). Mesoporous and adsorption behavior of algal biochar prepared via sequential hydrothermal carbonization and ZnCl_2_ activation. Bioresour. Technol..

[51] Liang H., Ding W., Zhang H., Peng F., Geng Z., She D., Li Y. (2022). A novel lignin-based hierarchical porous carbon for efficient and selective removal of Cr (VI) from wastewater. Int. J. Biol. Macromol..

[52] Liang H., Sun R., Song B., Sun Q., Peng P., She D. (2020). Preparation of nitrogen-doped porous carbon material by a hydrothermal-activation two-step method and its high-efficiency adsorption of Cr (VI). J. Hazard. Mater..

[53] Islam M.A., Ahmed M.J., Khanday W.A., Asif M., Hameed B.H. (2017). Mesoporous activated coconut shell-derived hydrochar prepared via hydrothermal carbonization-NaOH activation for methylene blue adsorption. J. Environ. Manag..

[54] Tan Y., Wang X., Xiong F., Ding J., Qing Y., Wu Y. (2021). Preparation of lignin-based porous carbon as an efficient absorbent for the removal of methylene blue. Ind. Crops Prod..

[55] Islam M.A., Benhouria A., Asif M., Hameed B.H. (2015). Methylene blue adsorption on factory-rejected tea activated carbon prepared by conjunction of hydrothermal carbonization and sodium hydroxide activation processes. J. Taiwan Inst. Chem. Eng..

[56] Islam M.A., Ahmed M.J., Khanday W.A., Asif M., Hameed B.H. (2017). Mesoporous activated carbon prepared from NaOH activation of rattan (*Lacosperma secundiflorum*) hydrochar for methylene blue removal. Ecotox. Environ. Saf..

[57] Bedin K.C., Martins A.C., Cazetta A.L., Pezoti O., Almeida V.C. (2016). KOH-activated carbon prepared from sucrose spherical carbon: Adsorption equilibrium, kinetic and thermodynamic studies for Methylene Blue removal. Chem. Eng. J..

[58] Tu W., Liu Y., Xie Z., Chen M., Ma L., Du G., Zhu M. (2021). A novel activation-hydrochar via hydrothermal carbonization and KOH activation of sewage sludge and coconut shell for biomass wastes: Preparation, characterization and adsorption properties. J. Colloid Interface Sci..

[59] Wang Y., Lu C., Cao X., Wang Q., Yang G., Chen J. (2022). Porous Carbon Spheres Derived from Hemicelluloses for Supercapacitor Application. Int. J. Mol. Sci..

[60] Song X., Gunawan P., Jiang R., Leong S., Wang K., Xu R. (2011). Surface activated carbon nanospheres for fast adsorption of silver ions from aqueous solutions. J. Hazard. Mater..

[61] Mumme J., Eckervogt L., Pielert J., Diakité M., Rupp F., Kern J. (2011). Hydrothermal carbonization of anaerobically digested maize silage. Bioresour. Technol..

[62] Koechermann J., Görsch K., Wirth B., Mühlenberg J., Klemm M. (2018). Hydrothermal carbonization: Temperature influence on hydrochar and aqueous phase composition during process water recirculation. J. Environ. Chem. Eng..

